# Immune Responses to Influenza D Virus in Calves Previously Infected with Bovine Viral Diarrhea Virus 2

**DOI:** 10.3390/v15122442

**Published:** 2023-12-16

**Authors:** Fernando Vicosa Bauermann, Shollie Falkenberg, Jennifer M. Rudd, Cristina Mendes Peter, Ingryd Merchioratto, Jerry W. Ritchey, John Gilliam, Jared Taylor, Hao Ma, Mayara Fernanda Maggioli

**Affiliations:** 1 Department of Veterinary Pathobiology, College of Veterinary Medicine, Oklahoma State University (OSU), Stillwater, OK 74078, USA; 2 Department of Pathobiology, College of Veterinary Medicine, Auburn University, Auburn, AL 36849, USA; 3 Animal Research Services, National Animal Disease Center, United States Department of Agriculture, Ames, IA 50010, USA; 4 Center for Medical Bioinformatics, Escola Paulista de Medicina, Federal University of Sao Paulo (UNIFESP), Sao Paulo 04039-032, Brazil; 5 Setor de Virologia, Departamento de Medicina Veterinária Preventiva, Universidade Federal de Santa Maria, Santa Maria 97105-900, Brazil; 6 Veterinary Clinical Sciences, College of Veterinary Medicine, Oklahoma State University, Stillwater, OK 74078, USA

**Keywords:** bovine viral diarrhea virus, immune responses, influenza D virus, pathogenesis, thymus

## Abstract

Bovine viral diarrhea virus (BVDV) induces immunosuppression and thymus depletion in calves. This study explores the impact of prior BVDV-2 exposure on the subsequent immune response to influenza D virus (IDV). Twenty 3-week-old calves were divided into four groups. Calves in G1 and G3 were mock-treated on day 0, while calves in G2 and G4 received BVDV. Calves in G1 (mock) and G2 (BVDV) were necropsied on day 13 post-infection. IDV was inoculated on day 21 in G3 calves (mock + IDV) and G4 (BVDV + IDV) and necropsy was conducted on day 42. Pre-exposed BVDV calves exhibited prolonged and increased IDV shedding in nasal secretions. An approximate 50% reduction in the thymus was observed in acutely infected BVDV calves (G2) compared to controls (G1). On day 42, thymus depletion was observed in two calves in G4, while three had normal weight. BVDV-2-exposed calves had impaired CD8 T cell proliferation after IDV recall stimulation, and the α/β T cell impairment was particularly evident in those with persistent thymic atrophy. Conversely, no difference in antibody levels against IDV was noted. BVDV-induced thymus depletion varied from transient to persistent. Persistent thymus atrophy was correlated with weaker T cell proliferation, suggesting correlation between persistent thymus atrophy and impaired T cell immune response to subsequent infections.

## 1. Introduction

Disorders of the respiratory tract account for significant economic losses for cattle producers worldwide. Most of these cases involve a combination of infectious agents and predisposing stressful conditions or environmental factors [1,2]. The list of pathogens involved in the bovine respiratory disease complex (BRDC) includes several viruses and bacteria. Among the viral pathogens are the bovine viral diarrhea virus (BVDV), bovine respiratory syncytial virus (BRSV), bovine herpesvirus type 1 (BoHV-1), bovine parainfluenza virus type 3 (PI-3), bovine coronavirus, bovine adenoviruses, bovine rhinoviruses, and the recently identified influenza D virus (IDV) [2,3,4].

IDV is frequently identified in cases of respiratory disease in cattle. However, the information about its role in the BRDC remains a matter of investigation [5,6]. Influenza D virus belongs to the *Orthomyxoviridae* family [7], *Deltainfluenzavirus* genus [8]. IDV was identified in 2011 in a 15-week-old pig displaying influenza-like clinical signs [6]. Although IDV was initially recognized in a swine with influenza-like clinical signs, surveillance studies indicated that bovines are likely the reservoir [9,10,11]. IDV pathogenesis studies in cattle are scarce, and the observed results diverge from mild disease restricted to the upper respiratory tract to moderate respiratory signs with involvement of the upper and lower respiratory tract [12,13,14,15,16].

BVDV is from the *Pestivirus* genus, *Flaviviridae* family. BVDV stands as one of the most critical BRDC pathogens and is highly prevalent worldwide. Despite the availability and wide use of BVDV vaccines in the US, BVDV is responsible for major economic losses for the beef and dairy cattle industry [1,2,17,18,19]. BVDV infection may lead to a vast range of clinical presentations in cattle, including respiratory and reproductive diseases [20]. The respiratory disease associated with BVDV infection usually occurs in calves younger than 9 months old and is rarely seen in adult animals [21]. Although severe respiratory disease due to BVDV infection is reported, the clinical signs are often restricted to transient relative lymphopenia and low-grade pyrexia that resolve within 3–10 days [22,23,24].

Still, the impact of BVDV infection goes beyond decreased reproductive performance and respiratory disease. Even though infection is typically mild, BVDV infection may increase the susceptibility to other pathogens due to its marked tropism for lymphoid tissues, leading to atrophy of the thymus, especially the cortical portion [23,24,25,26,27,28,29,30]. The thymus plays essential roles in the immune system [31], including the maturation of T cells on-site and the secretion of hormones and cytokines supporting lymphocyte differentiation [32]. Consequently, animals infected with BVDV, especially at a young age, may have prolonged altered immune responses to subsequent infections, enhancing the severity of secondary infections [33,34].

The thymus is a tissue frequently affected by infections. However, it possesses an extraordinary capacity of regeneration [32,35,36]. The fundamental mechanisms governing thymus regeneration are largely unknown [37]. Although the thymus is affected by infections across a variety of mammalian species, it typically repopulates within two weeks after disease resolution in children [38,39,40]. Comparable information is not available in cattle. Moreover, in some cases in children, regeneration may take several months or may only partially regenerate [41].

There is a scarcity of studies evaluating the immune responses to secondary pathogens following BVDV infection in calves. Therefore, to further understand the impact of BVDV on host responses to subsequent infections, this study investigated the pathogenesis and immune responses to IDV in calves previously exposed to BVDV.

## 2. Materials and Methods

### 2.1. Viruses and Cells

Primary bovine turbinate cells (BT) and swine testicle cells (ST) were, respectively, used to amplify the non-cytopathic (NCP) BVDV2 strain RS886 and the IDV isolate D/bovine/Texas/72/2017 strains. The BVDV2-RS886 was characterized in vitro and in vivo [22,24], and it was amplified and titrated in BT cells . The IDV D/bovine/Texas/72/2017 was isolated from a bovine respiratory sample, amplified, and titrated in swine testicle (ST) cells. The IDV isolate demonstrated low pathogenicity in calves during a previous study [16]. The cells employed for virus amplification were tested and free of BVDV following testing procedures previously described [42]. Cells were cultured at 37 °C with 5% CO_2_ in minimum essential medium (MEM) (Corning^®^, Mediatech, Inc., Manassas, VA, USA) supplemented with 10% fetal bovine serum (FBS; Seradigma^®^, VWR International, LLC, Radnor, PA, USA), 1% Antimycotic-Antibiotic 100× (Gibco^™^, Life Technologies, Grand Island, NY, USA), and gentamicin (50 μg/mL) (Corning). The cell culture medium (MEM) used for the amplification and titration of IDV was FBS-free and supplemented as described above, in addition to 0.1 μg/mL of TPCK trypsin (Pierce Biotechnology, ThermoFisher Scientific, Rockford, IL, USA) and 5% bovine serum albumin fraction V (Sigma-Aldrich, MilliporeSigma, Saint Louis, MO, USA).

### 2.2. Experimental BVDV and IDV Infection in Calves—Study Design

**Figure 1:**
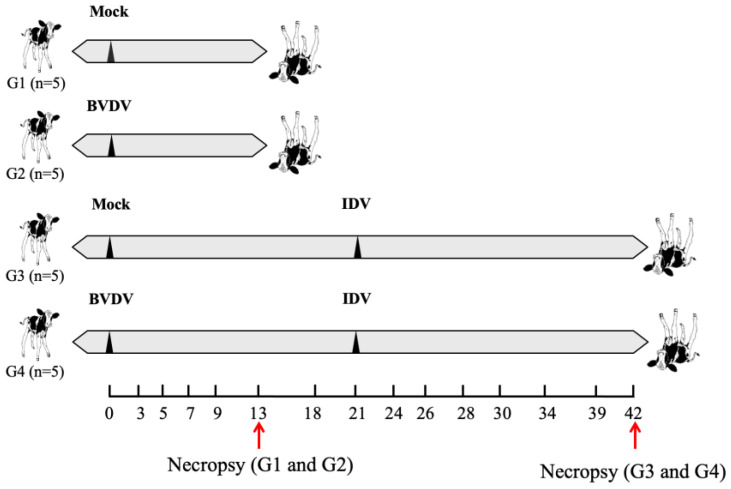
Experimental design. Animals in groups 1 (G1) and G3 were mock-inoculated on day 0, while animals in G2 and G4 were inoculated with BVDV. Inoculations are indicated by the black triangles in the horizontal bars representing the study timeline. Animals in G3 and G4 were inoculated with IDV on day 21. Necropsy, indicated by the red arrows, was conducted on days 13 (G1 and G2) and 42 (G3 and G4). Days with sample collection are marked in the study timeline, describing the specific days.

The animal study was conducted in an Animal Biosafety Level 2 (ABSL-2) facility at the College of Veterinary Medicine at Oklahoma State University. Animals were kept following the recommendations presented in the 4th edition of the Guide for the Care and Use of Agricultural Animals in Research and Teaching. The Oklahoma State University Institutional Animal Care and Use Committee approved the study, protocol number IACUC-20-14-STW. The study design is represented in Figure 1. Calves were separated from the dam after birth to avoid colostrum ingestion and passive transfer of antibodies against BVDV and influenza D virus. Mixed Holstein calves were tested and found free of BVDV and IDV antigens and antibodies. Twenty BVDV and IDV-naïve calves, about 3 weeks old, were divided into four groups. The animals in G2 and G4 were inoculated with BVDV2 via the nasal route with 5 mL (2.5 mL/nostril) of strain BVDV2-RS886 (titer 10^6^ TCID/mL) on day 0, while animals in G1 and G3 were mock-treated. G1 and G2 calves were necropsied past the BVDV acute phase on day 13 post-infection (pi) [26]. On day 21, the animals in G3 and G4 were inoculated intranasally with IDV strain D/bovine/Texas/72/2017 (titer 10^6^ TCID/mL) via the nasal route with 5 mL (2.5 mL/nostril). Animals in G3 and G4 were necropsied on day 42 post-BVDV inoculation (21 days post-IDV inoculation to allow seroconversion and T cell immune responses against IDV). The animals were monitored daily, and clinical signs were scored using the Calf Health Scorer system developed at the University of Wisconsin’s School of Veterinary Medicine. In brief, nasal discharge, ocular discharge, body temperature, behavior, feces, cough, and respiratory movements were monitored. Nasal swab, serum, and whole blood samples were collected on days 0, 3, 5, 7, 9, and 13 post-BVDV inoculation and on days 0, 3, 5, 7, 9, 13, 18, and 21 post-IDV inoculation (respectively, days 21, 24, 26, 28, 30, 34, 39, and 42 post-BVDV inoculation). Samples were used for monitoring viremia, virus shedding, and evaluation of the immune responses.

Nasal samples were collected using 15 cm sterile swabs. Each swab was used to sample both nostrils. The swabs were placed in microtubes containing 500 μL of PBS immediately after collection and then stored at −80 °C until use. Blood samples were collected by jugular venipuncture with Vacuette tubes containing a clot activator for serum separation (Greiner Bio-One, Monroe, NC, USA). Serum samples were aliquoted and stored at −20 °C until tested. Tubes containing heparin (Greiner Bio-One) were used to prepare peripheral blood mononuclear cells (PBMCs). PBMCs were separated using SepMate™ PBMC Isolation Tubes (STEMCELL Technologies, Cambridge, MA, USA) following the manufacturer’s recommendations. Purified PBMCs were eluted in a solution with 90% fetal bovine serum and 10% dimethylsulfoxide and cryopreserved in liquid nitrogen until testing.

The thymus was collected at necropsy, and the thymus weight was evaluated. A ratio of the thymus weight and the average weight of both kidneys (thymus/kidney ratio) was conducted to correct variations due to animal body size disparity [43].

### 2.3. BVDV and IDV RT-qPCR

Microtubes containing nasal swabs were thawed and vigorously vortexed for 15 s. The swab was removed, and the remaining fluid was used for RNA extraction. The RNA was extracted from nasal swab samples and serum samples using Quick-RNA Viral Kit (Zymo Research, Irvine, CA, USA) according to the manufacturer’s instructions. For the nucleic acid amplification, the BVDV primers 5′-TCTCGAGACTCCTCGACTAATG-3′ and 5′-TAGCAGTGAGTTCGTTGGATG-3′ were used with the probe 5′-/56-FAM/AGTGTCGAACCATTGACGACTCCC/36-TAMSp/-3′ (Integrated DNA Technologies, Coralville, IA, USA). The IDV amplification used primers 5′-CCAATGCTTCCTCCCTGTAAT-3′ and 5′-ATGGAGAGTGCTGCTTCTTG-3′ associated with the probe 5′-/56-FAM/AACATTCCCATCAGCATTCCTCCC/36-TAMSp/-3′ (IDT). The reactions were set up using Luna Universal Probe One-Step RT-qPCR Kit (New England Biolabs, Ipswich, MA, USA), according to the manufacturer’s instructions, and using the Applied Biosystems™ 7500 Fast Real-Time PCR System (Applied Biosystems, ThermoFisher Scientific, Foster City, CA, USA). Briefly, 20 μL reactions were prepared using 10 μL Luna Universal Probe One-Step Reaction Mix (2×), 2 μL of primer-probe mix 10× (PrimeTime qPCR assay, IDT), 1 μL of RNA (20 ng total), 1 μL of Luna WarmStart RT Enzyme Mix (20×), and nuclease-free water to complete the reaction volume. The thermocycling protocol consisted of reverse transcription for 10 min at 55 °C followed by 1 min denaturation at 95 °C and then 40 cycles of denaturation at 95 °C for 10 s and extension at 60 °C for 60 s.

### 2.4. Hemagglutination Inhibition (HI) and Virus Neutralization (VN) Assays

The HI and VN assays were conducted in serum samples collected prior to IDV inoculation and 21 days pi. VN was conducted using heat-inactivated (30 °C for 60 min) serum samples. The serum dilutions from 1:10 to 1:2560 were incubated with 200 TCID_50_ of IDV D/bovine/Texas/72/2017 or cytopathic BVDV-1 strain Singer for 60 min at 37 °C. The incubated serum samples were transferred to a 96-well plate containing a 90% confluent layer of ST cells for IDV VN and BT cells for BVDV VN testing. The plates were incubated for 4 days at 37 °C in 5% CO_2_. The presence of cytopathic effects was examined in the cells using a light microscope. The neutralizing antibody titers were measured by determining the highest serum dilution that completely prevented IDV or BVDV replication, expressed as the reciprocal value.

The HI assay was conducted following the methods previously outlined [6]. The serum samples were treated with Receptor Destroying Enzyme II (RDE II—Denka Seiken Co., Niigata-ken, Japan) at a 1:4 ratio for 18 h at 37 °C. Subsequently, the samples were heat-inactivated for 30 min at 56 °C and diluted to a final concentration of 1:10 in PBS. Two-fold serial dilutions were conducted and incubated with 4 agglutinating doses of IDV D/bovine/Texas/72/2017 for 1 h at room temperature. Following this, turkey red blood cells (Turkey Red Blood Cells Packed 5%, Innovative Research, Novi, MI, USA) at 0.5% were added. The presence of hemagglutination was examined, and the hemagglutination inhibition titers were determined by the highest serum dilution that completely prevented hemagglutination, expressed as the reciprocal value. Titers were transformed to geometric mean titers (GMT) as previously described [44].

### 2.5. Assessment of Cell-Mediated Immune Responses to IDV

IDV-specific cell-mediated responses elicited by PBMCs were assessed for bovine T cell subsets (CD4, CD8, γδ T cells) and included assessment of proliferation (CellTrace dilution assay) and IFN-γ expression after in vitro recall stimulation with IDV. To assess proliferation, PBMCs were stained with CellTrace violet 1 µg/mL (ThermoFisher Scientific), cultured in 96-well plates (1–5 × 10^5^ cells/well), and stimulated with live virus multiplicity of infection of 1 (MOI 1) or inactivated virus (by ultraviolet radiation) MOI 5 for 5 days at 37 °C. Pokeweed mitogen (PW) 10 µg/mL (Sigma-Aldrich) and RPMI 1640 media (Corning) were used as positive and negative controls, respectively. Following stimulation, proliferating T cells were determined using antibodies against T cell markers. Antigen-specific IFN-γ-secreting T cells in PBMCs were detected using intracellular cytokine staining (ICS) assays followed by flow cytometry analysis. For this, the cells were stimulated as described above, although cultivated for 18 h. The protein transport inhibitors brefeldin A (BD Biosciences, San Diego, CA, USA) and monensin (BD Biosciences) were added to the culture media during the last 8–12 h of incubation to prevent the release of cytokines by stimulated cells. Following incubation, cells were harvested and stained with antibodies against bovine lymphocyte subset markers and the appropriate secondary antibodies, followed by ICS using the CytoFix/CytoPerm fixation/permeabilization solution kit (BD Biosciences).

The antibody clones, anti-bovine markers, employed were as follows: CD3 (MIA11), CD4 (ILA11), CD8 (BAQ111A), TCR1 δ chain (GB21A) (Washington State University MAb Center, Pullman, WA, USA), and mouse anti-bovine IFN-γ-RPE (clone CC302, Bio-Rad). All proliferation and ICS flow cytometry assays used unstained cells, compensation, and fluorescence-minus-one (MFO) controls. LIVE/DEAD (BD Bioscience) fixable dye was included in the panels and used to exclude dead cells from the analysis. Cells were acquired using a BD LSR II (BD Biosciences) cytometer. At least 50,000 events were acquired, and the data were analyzed using FlowJo™ (BD Biosciences, Ashland, OR, USA). Differences in responding T cells were accounted for in each experimental group (using unstimulated samples to control for non-specific responses). A comparison between two groups (as well as intragroup) was conducted using Student’s *t*-test. Statistical evaluation was conducted using GraphPad Prism version 10 (Dotmatics, San Diego, CA, USA).

## 3. Results

### 3.1. Clinical Signs and Viral Detection

BVDV infection was confirmed in all animals based on at least one qPCR positive in nasal swabs and/or serum samples between days 0 and 18 pi (Table 1). Only mild and transient clinical signs were noted in animals inoculated with BVDV. Symptoms included inappetence, cough, and nasal and ocular discharge. The most consistent clinical finding was fever, especially on day 7, as detailed in Table 2. Following IDV inoculation, the highest number of animals presenting fever was on day 1 pi. No significant respiratory signs were noted in calves post-IDV inoculation.

**Figure 2:**
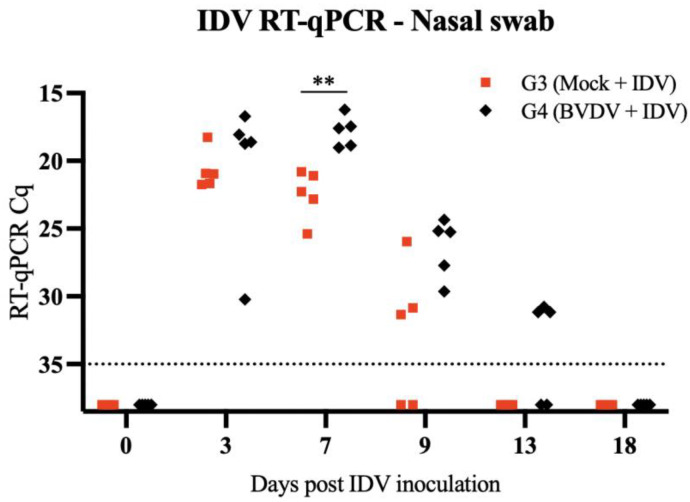
IDV RT-qPCR in nasal swab samples collected from calves in groups 3 and 4 (G3 and G4) following IDV inoculation (day 0 to day 18). Negative samples are represented below the dotted line that indicates the quantification cycle (Cq) 35. ** Denotes statistical significance *p* < 0.001.

The RT-qPCR performed on nasal swab samples demonstrated that animals pre-exposed to BVDV had prolonged IDV shedding and an increased concentration of IDV RNA. A significant difference in the shedding was noted on day 7 pi (Figure 2).

### 3.2. Thymus Evaluation

**Figure 3:**
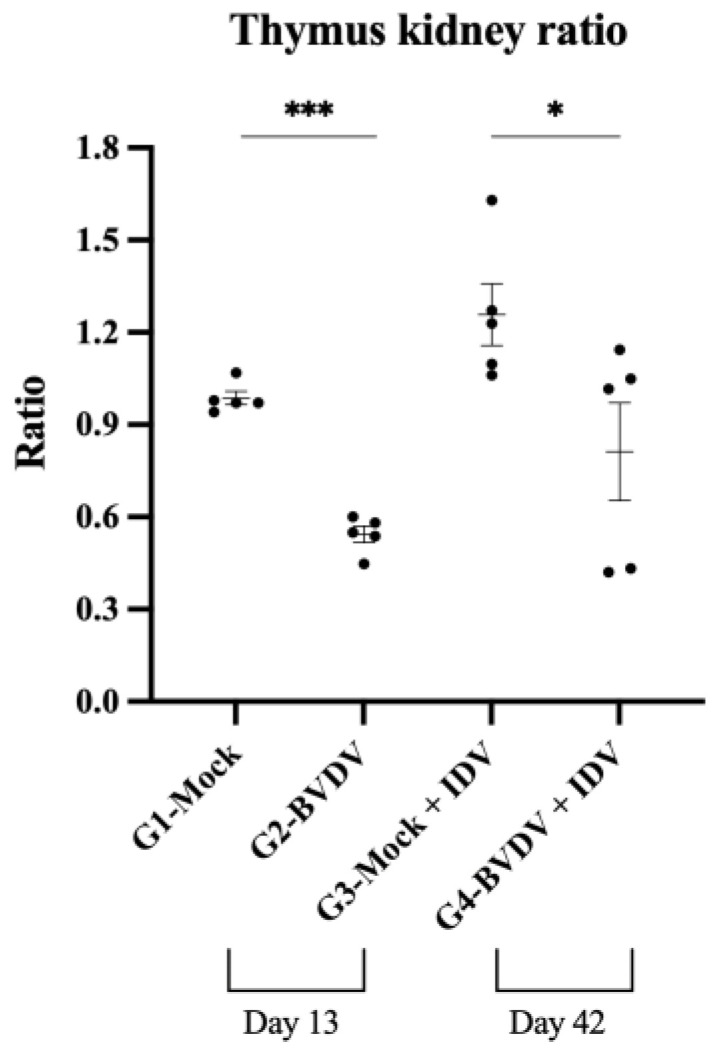
Corrected thymus weight (thymus/kidney ratio) in calves (represented by black dots) on days 13 (groups 1 and 2) and 42 post-BVDV inoculation (groups 3 and 4). * Denotes statistical significance *p* < 0.05 and *** denotes *p* < 0.0001.

The evaluation of the thymus weight demonstrated that all of the BVDV-infected animals (G2) had significant thymus atrophy (about 50% lower weight) on day 13 pi compared to the mock control group G1 (Figure 3). Interestingly, on day 21 post-IDV inoculation (BVDV day 42), three animals (60%) in G4 presented no thymus depletion, with weight comparable to the mock control group, G1. In contrast, thymus depletion was noted in two animals in G4. The two animals with persistent thymus atrophy had a thymus/kidney ratio comparable to animals in G2 (BVDV acute infection). No indication of thymus depletion was noted in animals in G3 (IDV-only group).

### 3.3. Cell-Mediated Immune Responses to IDV

**Figure 4:**
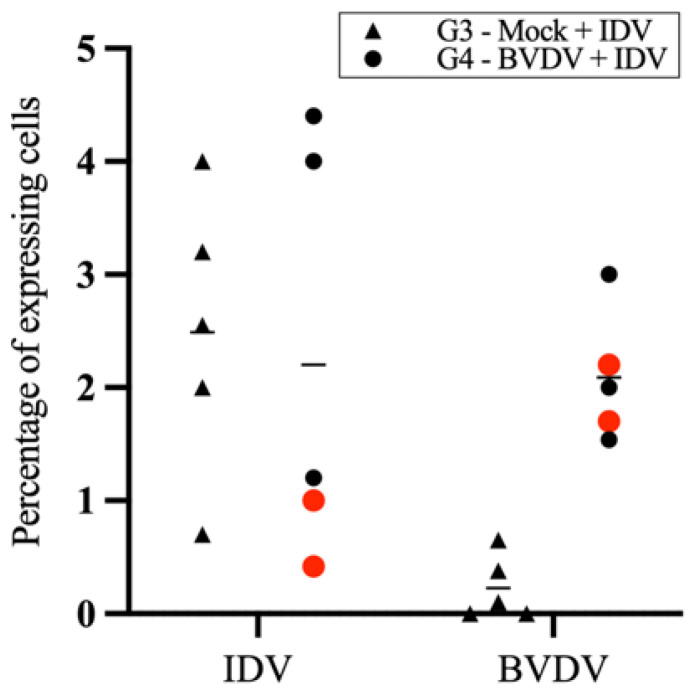
Percentage of cells expressing IFN-γ post-in vitro recall stimulation with IDV or BVDV in blood collected from calves 21 days post-IDV inoculation (BVDV, day 42). The animals represented by the red dots are the two animals with persistent thymus atrophy.

The immune responses following IDV recall stimulation using PBMCs collected on day 14 post-IDV inoculation showed two distinct subsets of animals in the group pre-inoculated with BVDV. The two animals with persistent thymus atrophy were among the three animals positive for IDV on day 13. Similarly, three (including the two with thymus atrophy, represented by red dots in Figure 4) showed a low number of interferon-gamma (IFN-γ)-expressing cells, while the other two calves had a considerably high percentage of IFN-γ-expressing cells (Figure 4). The difference between the subgroup composed of three animals with low levels of IFN-γ-expressing cells in the IDV G4 compared to the levels of IFN-γ-expressing cells in animals in IDV G3 was significant (*p* = 0.0019).

**Figure 5:**
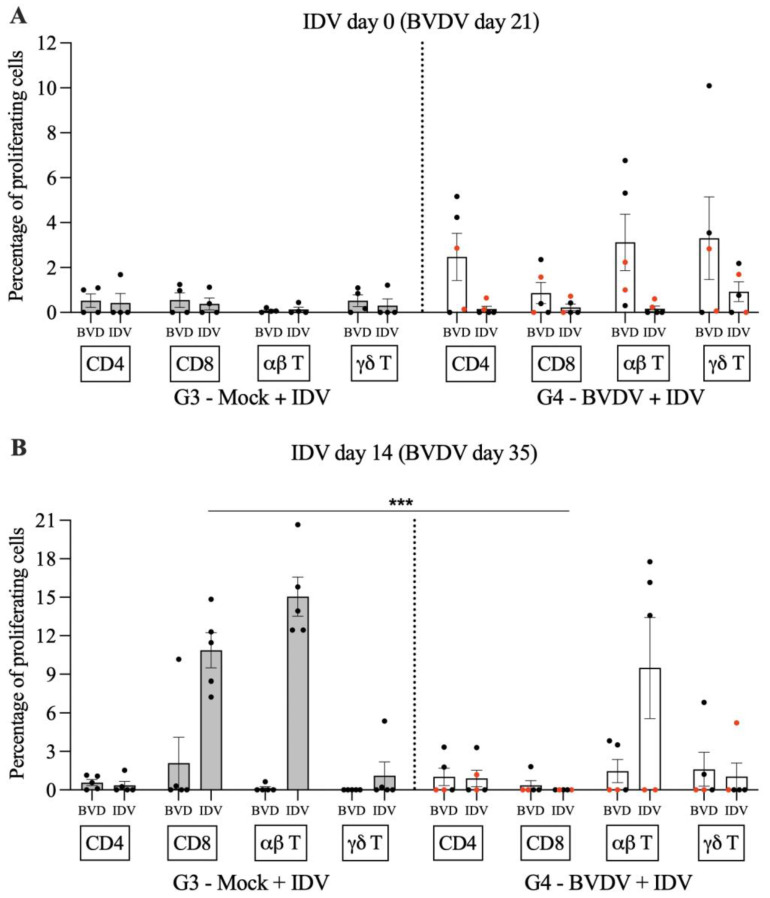
Percentage of proliferating lymphocytes following recall stimulation with BVDV and IDV on days 0 (**A**) and 14 (**B**) post-IDV infection (BVDV, days 21 and 35). Percentage of proliferating cell for animals in group 3 (G3) are represented by gray bars on the left side of the dotted line and by white bars for group 4 (G4) on the right side. The error bars indicate the standard error of the mean. The animals represented by the red dots are the two animals with persistent thymus atrophy. *** Denotes statistical significance *p* < 0.0001 for the IDV CD8 T cell comparison between animals in G3 and G4.

Following T cell recall stimulation with IDV antigens, it was verified that the proliferation of CD8 T cells was reduced in all animals pre-exposed to BVDV (Figure 5). Moreover, the two calves with persistent thymus atrophy had a significantly reduced proliferation of α/β T cells when compared to the three animals in G3 (*p* = 0.002) and also when compared to the three animals in G4 that apparently recovered from the thymus atrophy (*p* = 0.0021).

### 3.4. Serological Responses to BVDV and IDV

**Figure 6:**
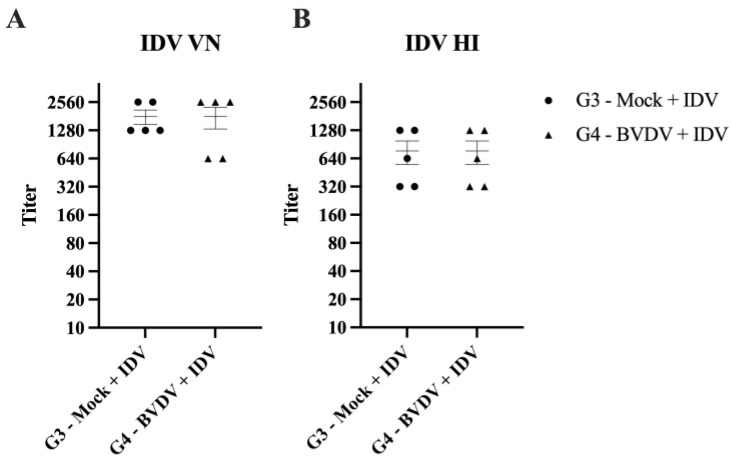
Serology to IDV. The antibody response to IDV was evaluated on serum samples collected 21 days post-IDV inoculation (BVDV, day 42). (**A**) Neutralizing antibody titers for IDV assessed by virus neutralization (VN) test. (**B**) Antibody levels for IDV assessed by hemagglutination inhibition (HI) assay. The error bars indicate the standard error of the mean.

VN for BVDV revealed that all five animals in G4 had titers against BVDV (titers: 80, 160, 160, 320, and 320) on day 42 of the study. In contrast, animals in G2 had no detectable titers (<10) of neutralizing antibodies against BVDV in samples collected during necropsy on day 13. Animals in G1 and G3 were also negative for antibodies against BVDV on samples collected during necropsy (days 13 and day 42, respectively). Serology against IDV demonstrates detectable titers only in animals from G3 and G4. The serum titers retrieved in the VN and HI assays are presented in Figure 6. No significant difference in titers to IDV was noted, independent of previous BVDV infection. The GMT for the VN was 1688 for G3 and 1470 for G4. The GMT for HI was 640 for both groups.

## 4. Discussion

BVDV infection is recognized for its capacity to enhance susceptibility to subsequent secondary respiratory infections due to a transient state of immunosuppression [2,4,22,29,45,46]. Under experimental conditions, it was demonstrated that calves infected with BVDV and subsequently infected with BoHV-1 exhibited more pronounced histopathological alterations compared to calves solely subjected to BoHV-1 inoculation [34]. In the present investigation, the utilization of the low pathogenic isolate of IDV yielded no discernible differences in clinical presentation when compared against pre-exposed calves or those not previously exposed to BVDV-2. Only brief episodes of fever were observed, accompanied by the absence of respiratory signs.

A previous study administered IDV via aerosol inhalation to colostrum-deprived calves, which resulted in the manifestation of mild to moderate clinical signs marked by upper and lower respiratory tract involvement [13]. Conversely, alternate studies employing intranasal IDV instillation to infect calves resulted in either asymptomatic infection or mild clinical signs confined to the upper respiratory tract [12,14]. In addition to the inherent variability in pathogenicity among distinct isolates, the methodology of inoculation is also assumed to exert an influence on the severity of disease manifestations. It is important to note that the timing of the secondary infection, post-BVDV-2, also plays a pivotal role in the enhanced clinical presentation, as described during a dual-infection study encompassing BVDV and bovine coronavirus [4].

Our findings present evidence indicating that subsequent to BVDV-2 infection, a subset of the examined calves (60%) exhibited a recovery from BVDV-induced thymus atrophy within a span of 6 weeks post-BVDV-2 challenge (day 42), whereas another subset of calves (40%) demonstrated either delayed recuperation or sustained damage. Hematopoietic organs and cells of the immune system commonly serve as targets for viral infections [32,47]. Bovine viral diarrhea virus (BVDV) stands among the viruses known to induce impairment and dysfunction of the thymus [22,23,28,30].

The thymus holds a central position within the immune system [48], playing a pivotal role in the on-site maturation of T cells and in the secretion of hormones and cytokines that bolster lymphocyte differentiation [48,49,50]. Previous investigations have extensively explored the BVDV-induced histopathological alterations within the thymus of calves, outlining a distinctive reduction in the cortex-to-medulla ratio. This diminution has been attributed to medullary thickening and concurrent cortex depletion [23,28,30]. The observed thymus atrophy results from lymphocyte depletion as a consequence of thymocyte apoptosis and the disruption of thymocyte development processes [32]. The thymus involution during infection is also attributed, at least partially, to the increased levels of TNFα and IFNγ [37].

The restoration of an immunocompetent state following instances of immunosuppression is of paramount importance for the effective clearance of subsequent infections by the host. Calves exhibiting persistent thymus depletion coincided with compromised proliferation of α/β T cells, as compared to those calves ostensibly recuperated from thymus injury. Interestingly, among the animals in G4, two subgroups were evidently related to the percentage of cells expressing IFN-γ. Three calves (two with persistent thymus atrophy and one calf that apparently recovered from thymus atrophy) had lower levels of IFN-γ-expressing cells compared to the other two calves in the group. This impairment in IFN-γ response was also observed in calves pre-exposed to BVDV during subsequent BoHV-1 infection [34]. In addition, that study also evidenced intense inflammatory responses associated with increased secretion of TNFα (proinflammatory cytokine) but decreased levels of IL-10 (anti-inflammatory cytokine) in BVDV-exposed calves [34].

Despite the fact that some animals may partially recover from thymus depletion through tissue remodeling and collagen deposition [33], complete restoration of thymus function, assessed by intracellular cytokine staining after recall stimulation, might not be achieved. This might explain why one calf that apparently recovered from the thymus atrophy had levels of IFN-γ-expressing cells comparable with the two calves with persistent thymus atrophy.

Irrespective of persistent thymus atrophy, all calves infected with BVDV later exhibited compromised CD8 responses subsequent to IDV recall stimulation. The immune response impairment was further corroborated by notably elevated and prolonged shedding of IDV in nasal secretions in calves pre-exposed to BVDV. Although the pathogenicity of BVDV may vary between strains, studies have shown that peripheral CD8 cells are the most impacted during BVDV infection, followed by CD4 [51,52,53]. Conversely, the impact on circulating gamma-delta T cells (γ/δ T cells) was limited in previous studies [51,52,54]. These findings are in agreement with previous studies that have demonstrated the quicker recovery and milder effect of BVDV on CD4 T cells in calves [23,28]. Additionally, it is noteworthy that the BVDV-2 infection or prolonged thymus atrophy does not impede humoral responses directed toward IDV. The comparable levels of neutralizing antibodies and the hemagglutination inhibition titers against IDV evidence this.

In summary, our findings indicate that BVDV-induced thymic injury might result in permanent consequences for calves’ immunity. Although certain animals apparently recovered from the thymus depletion, the short- and long-term implications for thymic function remain largely unknown. Notably, our results highlight that those calves previously infected with BVDV-2 (irrespective of thymus regeneration) exhibited a compromised CD8 response upon subsequent infection with IDV. Furthermore, the lower proliferation of αβ T cells in calves that recuperated from BVDV-2 infection (assessed by the lack of shedding, viremia, and clinical signs) yet sustained thymus atrophy demonstrates immune impairment in comparison to calves that recovered from thymus depletion. In contrast, the levels of neutralizing antibodies against IDV remained unchanged, irrespective of the presence of thymus atrophy or prior BVDV-2 infection. Conducting a more extensive investigation to enhance our understanding of the disruptions in thymic function triggered by BVDV and their subsequent influence on shaping modified immunity is of utmost importance. This holds particular significance in young individuals, as they exhibit the highest rates of T cell generation and are actively refining their T cell repertoires.

## Figures and Tables

**Table 1:** Number of animals presenting positive BVDV RT-qPCR in nasal swab and serum samples. Samples were tested on days 0, 3, 7, 9, 13, and 18 (D0, D3, D7, D9, D13, and D18) following BVDV-2 inoculation. No positive BVDV RT-qPCR was retrieved from animals in groups 1 and 3.

Number of Animals with Positive BVDV RT-qPCR	Sample Type	D0	D3	D7	D9	D13	D18
G2 BVDV	Nasal swab	0	0	5	4	0	NA *
	Serum	0	0	1	5	0	NA
G4 BVDV + IDV	Nasal swab	0	2	5	5	0	0
	Serum	0	0	2	3	0	0

* No sample available due to euthanasia conducted on day 13.

**Table 2:** Number of animals presenting fever (temperature above 39 °C) after BVDV or IDV inoculation between day 1 (D1) and day 8 (D8). No fever was noted in animals in control group 1.

Number of Animals Presenting Fever		D1	D2	D3	D4	D5	D6	D7	D8
Post-BVDV inoculation	G2 BVDV	-	2	-	1	-	3	4	1
	G4 BVDV + IDV	-	-	2	-	2	2	5	2
Post-IDV inoculation	G3 Mock + IDV	1	1	-	-	-	-	1	-
	G4 BVDV + IDV	3	-	-	-	1	-	-	-

## Data Availability

The data presented in this study are available on request from the corresponding author.

## References

[1] USDA-APHIS (2017). Death Loss in U.S. Cattle and Calves Due to Predator and Nonpredator Causes, 2015.

[2] Taylor J.D., Fulton R.W., Lehenbauer T.W., Step D.L., Confer A.W. (2010). The epidemiology of bovine respiratory disease: What is the evidence for predisposing factors?. Can. Vet. J..

[3] Fulton R.W. (2020). Viruses in Bovine Respiratory Disease in North America: Knowledge Advances Using Genomic Testing. Vet. Clin. N. Am. Food Anim. Pract..

[4] Ridpath J.F., Fulton R.W., Bauermann F.V., Falkenberg S.M., Welch J., Confer A.W. (2020). Sequential exposure to bovine viral diarrhea virus and bovine coronavirus results in increased respiratory disease lesions: Clinical, immunologic, pathologic, and immunohistochemical findings. J. Vet. Diagn. Investig. Off. Publ. Am. Assoc. Vet. Lab. Diagn. Inc.

[5] Ng T.F.F., Kondov N.O., Deng X., Van Eenennaam A., Neibergs H.L., Delwart E. (2015). A Metagenomics and Case-Control Study To Identify Viruses Associated with Bovine Respiratory Disease. J. Virol..

[6] Hause B.M., Ducatez M., Collin E.A., Ran Z., Liu R., Sheng Z., Armien A., Kaplan B., Chakravarty S., Hoppe A.D. (2013). Isolation of a Novel Swine Influenza Virus from Oklahoma in 2011 Which Is Distantly Related to Human Influenza C Viruses. PLoS Pathog..

[7] Shaw M., Palese P., Knipe D., Howley P. (2013). Fields Virology.

[8] Hause B.M., Collin E.A., Liu R., Huang B., Sheng Z., Lu W., Wang D., Nelson E.A., Li F. (2014). Characterization of a novel influenza virus in cattle and swine: Proposal for a new genus in the Orthomyxoviridae family. MBio.

[9] Ferguson L., Eckard L., Epperson W.B., Long L.P., Smith D., Huston C., Genova S., Webby R., Wan X.F. (2015). Influenza D virus infection in Mississippi beef cattle. Virology.

[10] Su S., Fu X., Li G., Kerlin F., Veit M. (2017). Novel Influenza D virus: Epidemiology, pathology, evolution and biological characteristics. Virulence.

[11] White S.K., Ma W., McDaniel C.J., Gray G.C., Lednicky J.A. (2016). Serologic evidence of exposure to influenza D virus among persons with occupational contact with cattle. J. Clin. Virol..

[12] Ferguson L., Olivier A.K., Genova S., Epperson W.B., Smith D.R., Schneider L., Barton K., McCuan K., Webby R.J., Wan X.-F. (2016). Pathogenesis of Influenza D Virus in Cattle. J. Virol..

[13] Cassard H., Ducatez M., Valarcher J.-F., Pinard A., Näslund K., Foret C., Hägglund S., Salem E., Corre T., Meyer G. (2019). Pathogenesis, host innate immune response and aerosol transmission of Influenza D virus in cattle. J. Virol..

[14] Hause B.M., Huntimer L., Falkenberg S., Henningson J., Lechtenberg K., Halbur T. (2017). An inactivated influenza D virus vaccine partially protects cattle from respiratory disease caused by homologous challenge. Vet. Microbiol..

[15] Bauermann F.V., Falkenberg S.M., Decaro N., Flores E.F., Ridpath J.F. (2015). Experimental infection of calves, sheep, goats and pigs with HoBi-like viruses by direct inoculation or exposure to persistently infected calves. Vet. Microbiol..

[16] Kaplan B.S., Falkenberg S., Dassanayake R., Neill J., Velayudhan B., Li F., Vincent A.L. (2021). Virus strain influenced the interspecies transmission of influenza D virus between calves and pigs. Transbound. Emerg. Dis..

[17] Ridpath J.F., Bolin S.R., Dubovi E.J. (1994). Segregation of Bovine Viral Diarrhea Virus into Genotypes. Virology.

[18] Houe H. (2003). Economic impact of BVDV infection in dairies. Biologicals.

[19] Larson R.L. (2015). Bovine Viral Diarrhea Virus–Associated Disease in Feedlot Cattle. Vet. Clin. N. Am. Food Anim. Pract..

[20] Baker J.C. (1995). The clinical manifestations of bovine viral diarrhea infection. Vet. Clin. N. Am. Food Anim. Pract..

[21] Baker J.C. (1987). Bovine viral diarrhea virus: A review. J. Am. Vet. Med. Assoc..

[22] Ridpath J.F., Falkenberg S.M., Bauermann F.V., VanderLey B.L., Do Y., Flores E.F., Rodman D.M., Neill J.D. (2013). Comparison of acute infection of calves exposed to a high-virulence or low-virulence bovine viral diarrhea virus or a HoBi-like virus. Am. J. Vet. Res..

[23] Falkenberg S.M., Bauermann F.V., Ridpath J.F. (2017). Characterization of thymus-associated lymphoid depletion in bovine calves acutely or persistently infected with bovine viral diarrhea virus 1, bovine viral diarrhea virus 2 or HoBi-like pestivirus. Arch. Virol..

[24] Liebler-Tenorio E.M., Ridpath J.F., Neill J.D. (2003). Distribution of viral antigen and development of lesions after experimental infection of calves with a BVDV 2 strain of low virulence. J. Vet. Diagn. Investig..

[25] Liebler-Tenorio E.M., Greiser-Wilke I., Pohlenz J.F. (1997). Organ and tissue distribution of the antigen of the cytopathogenie bovine virus diarrhea virus in the early and advanced phase of experimental mucosal disease. Arch. Virol..

[26] Liebler-Tenorio E.M., Ridpath J.F., Neill J.D. (2004). Distribution of viral antigen and tissue lesions in persistent and acute infection with the homologous strain of noncytopathic bovine viral diarrhea virus. J. Vet. Diagn. Investig..

[27] Frink S., Grummer B., Pohlenz J.F., Liebler-Tenorio E.M. (2002). Changes in distribution and numbers of CD4+ and CD8+ T-lymphocytes in lymphoid tissues and intestinal mucosa in the early phase of experimentally induced early onset mucosal disease in cattle. J. Vet. Med. Ser. B.

[28] Falkenberg S.M., Johnson C., Bauermann F.V., McGill J., Palmer M.V., Sacco R.E., Ridpath J.F. (2014). Changes observed in the thymus and lymph nodes 14 days after exposure to BVDV field strains of enhanced or typical virulence in neonatal calves. Vet. Immunol. Immunopathol..

[29] Risalde M.A., Molina V., Sánchez-Cordón P.J., Pedrera M., Romero-Palomo F., Bautista M.J., Moreno A., Gómez-Villamandos J.C. (2013). Comparison of pathological changes and viral antigen distribution in tissues of calves with and without preexisting bovine viral diarrhea virus infection following challenge with bovine herpesvirus-1. Am. J. Vet. Res..

[30] Romero-Palomo F., Risalde M.A., Gómez-Villamandos J.C. (2017). Immunopathologic Changes in the Thymus of Calves Pre-infected with BVDV and Challenged with BHV-1. Transbound. Emerg. Dis..

[31] Cunningham C.P., Kimpton W.G., Holder J.E., Cahill R.N.P. (2001). Thymic export in aged sheep: A continuous role for the thymus throughout pre- and postnatal life. Eur. J. Immunol..

[32] Savino W. (2006). The thymus is a common target organ in infectious diseases. PLoS Pathog..

[33] Romero-Palomo F., Risalde M.A., Molina V., Lauzi S., Bautista M.J., Gómez-Villamandos J.C. (2015). Characterization of thymus atrophy in calves with subclinical BVD challenged with BHV-1. Vet. Microbiol..

[34] Risalde M.A., Molina V., Sánchez-Cordón P.J., Pedrera M., Panadero R., Romero-Palomo F., Gómez-Villamandos J.C. (2011). Response of proinflammatory and anti-inflammatory cytokines in calves with subclinical bovine viral diarrhea challenged with bovine herpesvirus-1. Vet. Immunol. Immunopathol..

[35] Dudakov J.A., Hanash A.M., Jenq R.R., Young L.F., Ghosh A., Singer N.V., West M.L., Smith O.M., Holland A.M., Tsai J.J. (2012). Interleukin-22 drives endogenous thymic regeneration in mice. Science.

[36] van den Broek T., Delemarre E.M., Janssen W.J.M., Nievelstein R.A.J., Broen J.C., Tesselaar K., Borghans J.A.M., Nieuwenhuis E.E.S., Prakken B.J., Mokry M. (2016). Neonatal thymectomy reveals differentiation and plasticity within human naive T cells. J. Clin. Investig..

[37] Granadier D., Iovino L., Kinsella S., Dudakov J.A. (2021). Dynamics of thymus function and T cell receptor repertoire breadth in health and disease. Semin. Immunopathol..

[38] Ross E.A., Coughlan R.E., Flores-Langarica A., Lax S., Nicholson J., Desanti G.E., Marshall J.L., Bobat S., Hitchcock J., White A. (2012). Thymic function is maintained during Salmonella-induced atrophy and recovery. J. Immunol..

[39] Anz D., Thaler R., Stephan N., Waibler Z., Trauscheid M.J., Scholz C., Kalinke U., Barchet W., Endres S., Bourquin C. (2009). Activation of melanoma differentiation-associated gene 5 causes rapid involution of the thymus. J. Immunol..

[40] Velardi E., Tsai J.J., van den Brink M.R.M. (2021). T cell regeneration after immunological injury. Nat. Rev. Immunol..

[41] Mackall C.L., Fleisher T.A., Brown M.R., Andrich M.P., Chen C.C., Feuerstein I.M., Horowitz M.E., Magrath I.T., Shad A.T., Steinberg S.M. (1995). Age, Thymopoiesis, and CD4+ T-Lymphocyte Regeneration after Intensive Chemotherapy. N. Engl. J. Med..

[42] Bauermann F.V., Flores E.F., Falkenberg S.M., Weiblen R., Ridpath J.F. (2014). Lack of evidence for the presence of emerging HoBi-like viruses in North American fetal bovine serum lots. J. Vet. Diagn. Investig..

[43] Kraybill H.F., Hiner R.L., Farnworth V.M. (1954). The Relation of Organ Weights to Lean Body Mass and Empty Body Weight in Cattle. J. Anim. Sci..

[44] Thrusfield M., Thrusfield M., Christley R. (2018). Veterinary Epidemiologyle. Veterinary Epidemiology.

[45] Rebhun W.C., French T.W., Perdrizet J.A., Dubovi E.J., Dill S.G., Karcher L.F. (1989). Thrombocytopenia associated with acute bovine virus diarrhea infection in cattle. J. Vet. Intern. Med..

[46] Bolin S.R., McClurkin A.W., Coria M.F. (1985). Effects of bovine viral diarrhea virus on the percentages and absolute numbers of circulating 3 and T lymphocytes in cattle. Am. J. Vet. Res..

[47] Messias C.V., Loss-Morais G., Carvalho J.B.d., González M.N., Cunha D.P., Vasconcelos Z., Arge L.W.P., Farias-de-Oliveira D.A., Gerber A.L., Portari E.A. (2020). Zika virus targets the human thymic epithelium. Sci. Rep..

[48] Thapa P., Farber D.L. (2019). The Role of the Thymus in the Immune Response. Thorac. Surg. Clin..

[49] Martinez-Ruíz G.U., Morales-Sánchez A., Bhandoola A. (2022). Transcriptional and epigenetic regulation in thymic epithelial cells. Immunol. Rev..

[50] Kurd N., Robey E.A. (2016). T-cell selection in the thymus: A spatial and temporal perspective. Immunol. Rev..

[51] Ellis J.A., Davis W.C., Belden E.L., Pratt D.L. (1988). Flow cytofluorimetric analysis of lymphocyte subset alterations in cattle infected with bovine viral diarrhea virus. Vet. Pathol..

[52] Brodersen B.W., Kelling C.L. (1999). Alteration of leukocyte populations in calves concurrently infected with bovine respiratory syncytial virus and bovine viral diarrhea virus. Viral Immunol..

[53] Gånheim C., Johannisson A., Ohagen P., Persson Waller K. (2005). Changes in peripheral blood leucocyte counts and subpopulations after experimental infection with BVDV and/or Mannheimia haemolytica. J. Vet. Med. B Infect. Dis. Vet. Public Health.

[54] Chase C.C.L. (2013). The impact of BVDV infection on adaptive immunity. Biologicals.

